# Effects of bisphosphonates in preventing periprosthetic bone loss following total hip arthroplasty: a systematic review and meta-analysis

**DOI:** 10.1186/s13018-018-0918-7

**Published:** 2018-09-04

**Authors:** Jialing Shi, Guang Liang, Rongzhi Huang, Liang Liao, Danlu Qin

**Affiliations:** 1 Guangxi Medical University, No. 22, Shuang Yong Road, Nanning, 530021 Guangxi Zhuang Autonomous Region China; 2 The first affiliated Hospital of Guangxi Medical University, The First Clinical Medical College, No. 6, Shuang Yong Road, Nanning, 530021 Guangxi Zhuang Autonomous Region China; 3 Department of the Second Endocrinology Ward, Jiangbin Hospital of Guangxi Zhuang Autonomous Region, Nanning, 530021 Guangxi Zhuang Autonomous Region China

**Keywords:** Bisphosphonates, Total hip arthroplasty, Bone resorption, Meta-analysis

## Abstract

**Background:**

Periprosthetic bone loss following total hip arthroplasty (THA) was a well-known phenomenon. This systematic review was to assess the effectiveness of bisphosphonates (BPs) for decreasing periprosthetic bone resorption.

**Methods:**

The MEDLINE, EMBASE, and Cochrane Library databases were searched up to March 2018. Randomized controlled trials compared the effects between administrating BPs and placebo or no medication were eligible; the target participants were patients who underwent THA. Mean differences (MD) and 95% confidence interval (95% CI) were calculated by using the random-effects models. Statistical analyses were performed by RevMan 5.3 software.

**Results:**

Fourteen trials involving 620 patients underwent THA were retrieved. BPs significantly prevented the loss of periprosthetic bone mineral density at 1 year (MD, 0.06 [95% CI, 0.03 to 0.08], *p* < 0.001), between 2 and 4 years (MD, 0.04 [95% CI, 0.01 to 0.07], *p =* 0.02), and more than 5 years after THA (MD, 0.08 [95% CI, 0.06 to 0.11], *p* < 0.001). Both serum bone alkaline phosphatase (MD, − 7.28 [95% CI, − 9.81 to − 4.75], *p* < 0.001) and urinary N-telopeptide of type I collagen (MD, − 24.37 [95% CI, − 36.37 to − 12.37], *p* < 0.001) in BP group were significantly lower. Subgroup analyses showed that the third-generation BPs were more effective in decreasing periprosthetic bone loss than the first and second generation within 1 year after THA (*p* = 0.001).

**Conclusion:**

BPs were beneficial to decreasing periprosthetic bone loss. The third-generation BPs showed significantly efficacy for patients in short-term observation.

## Background

Total hip arthroplasty (THA) has become the most effective therapy for severe osteoarthritis [1–3]. It was estimated that approximately 572,000 patients will demand primary THA in the USA by the year 2030 [4]. Periprosthetic bone resorption following THA was a well-known phenomenon [5]. It may increase late-occurring periprosthetic fractures [6]. Moreover, bone resorption may decrease the primary stability of the implant and lead to progressive implant loosening [7], which was considered as the most common reason for revision [8]. Compared with primary THA, the risk of local and systemic complications increased and favorable benefits decreased in revision surgeries [9]. Therefore, strategies for inhibiting periprosthetic bone resorption and maintaining bone stock were essential.

Bisphosphonates (BPs), a family of drugs with a strong anti-osteoclast activity, were widely used for the first-line treatment of osteoporosis [10]. Mass data had showed that BPs inhibited bone resorption, increased bone mineral density, and reduced the risk of fractures [11]. Nevertheless, there was still controversy about the effect and mechanism of BPs on inhibiting periprosthetic bone loss after THA. Some studies indicated that BPs had no significant effect on suppressing bone loss after THA [12, 13]. In contrast, previous meta-analyses suggested BPs could inhibit early bone resorption around the implant [14–17]. However, these studies only included randomized controlled trials (RCTs) published before 2011. And target participants were not only THA but also total knee arthroplasty (TKA) and hemiarthroplasty in some studies. Compared with previous articles, this meta-analysis complemented the latest RCTs and had a larger sample size (620 patients). Moreover, it applied more rigorous eligibility of criteria and excluded trials involving TKA or hemiarthroplasty to reduce heterogeneity.

It was essential to perform a meta-analysis based on the latest evidence. This systematic review was to assess the effectiveness of BPs for decreasing periprosthetic bone resorption.

## Methods

### Literature search

The electronic literature search lasted up to 10 March 2018. Without language restrictions, reviewers searched PubMed (1966 to present), EMBASE (1980 to present), Ovid (1860 to present), and the Cochrane Library (Issue 1, 2017) by using the following items: “total hip arthroplasty,” “bisphosphonates,” “bone resorption,” and their associated words. Reference lists of all the selected studies were hand-searched for any additional trials. Two reviewers independently assessed trials for inclusion and resolved disagreements by discussion.

### Inclusion and exclusion criteria

Studies were eligible for inclusion: (1) target participants were patients who underwent THA, (2) compared the effects between administrating BPs and placebo or no medication, and (3) randomized controlled trials. We excluded studies if (1) participants had a history of metabolic bone diseases, bone tumor, or renal failure; (2) the same randomized controlled trial was reanalyzed with a shorter follow-up.

### Outcome measure

The primary outcome was periprosthetic bone mineral density (BMD) because this data is the most intuitive index to reflect the extent of periprosthetic bone loss. In order to analyze the bone turnover activity, researchers also collected the data of biochemical bone turnover (serum bone alkaline phosphates (BAP, U/L) and urinary N-telopeptide of type I collagen (NTX-I, nmol/mmol Cr)) as the second outcome.

### Quality assessment

Two reviewers independently assessed quality. Quality assessment consisted of random sequence generation, allocation concealment, blinding, incomplete outcome data, selective reporting, and other potential bias.

### Data extraction

The data was extracted in table that included the first author, year of publication, original country, primary disease, type of THA, type of BPs, control group, the number of participants, treatment duration, time of following, and the number of loss to follow-up. If the data was not reported in the text or the table in the article, it was extrapolated from the accompanying graphs. Reviewers asked the corresponding author of the eligible study for additional information when necessary.

### Statistical analysis

Statistical analysis was performed using Review Manager 5.3. Mean differences (MD) and 95% confidence interval (95% CI) were calculated for continuous outcomes. Meta-analysis was done according to a random-effects model. *P* < 0.05 was considered statistically significant. Heterogeneity was tested by using chi-square test with significance being set at *p* > 0.1 and *I*-square (*I*^2^) was used to estimate total variation across studies due to heterogeneity in percentage. *I*^2^ greater than 50% was considered as denoting substantial heterogeneity.

## Result

### Study identification

**Fig. 1:**
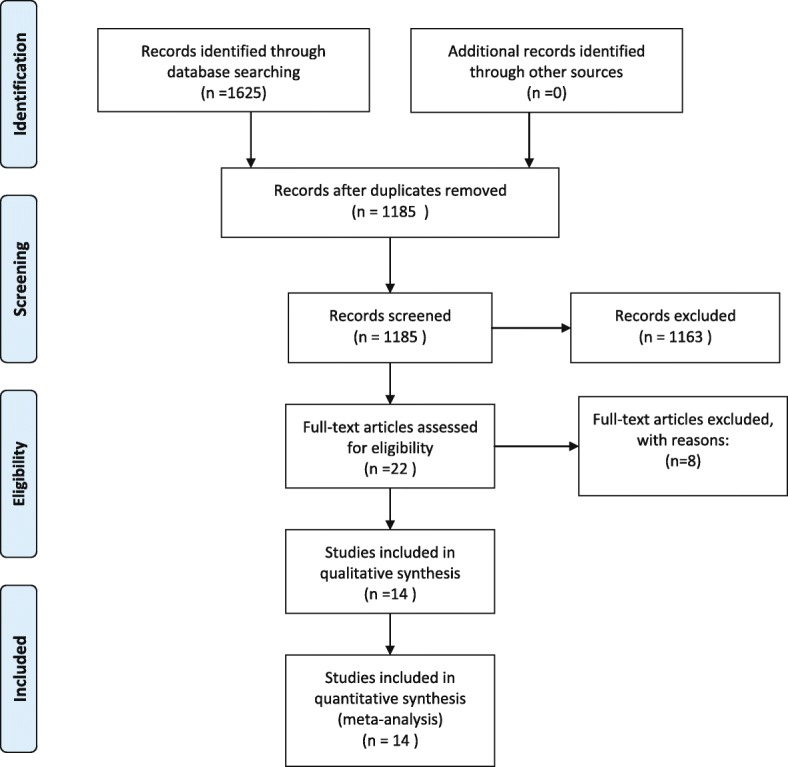
Flow diagram of included studies

The search identified 1625 potentially relevant references. Four hundred forty trials were excluded for duplicates. And 1185 trials were eliminated all based on titles and abstracts but 22 trials. After requiring full-text review, 14 trials met the inclusion criteria. Eight trials were excluded for several reasons: participants underwent hemiarthroplasty (two trials), shorter follow-up and reanalyzed data (three trials), and shared groups of participants because participants, authors, and designs were similar (three trials). The rest of 14 trials were included in qualitative synthesis. Finally, these 14 trials published from 2004 to 2017 were included in our systematic review [18–31] (Fig. 1).

### Characteristic of the studies

The included 14 studies were published from 2004 to 2017, with 318 participants receiving BPs and 302 receiving placebo or no medication. Table 1 provided more detailed information on these studies. Types of BPs were consisted of alendronate (six trials), etidronate (two trials), risedronate (three trials), pamidronate (one trial), zoledronate (one trial), and clodronate (one trial). G 1–2 BPs (etidronate, clodronate, and pamidronate) have simple R2 side chains. And G 3 BPs (alendronate, risedronate, and zoledronate) were developed by modifying the R2 side chain to include an amino group and heterocyclic structures. The dose and the duration of BP administration were different among the studies. The sample size ranged from 16 to 91 patients. Eleven trials reported BMD at different time points after THA surgery (ranged from 24 weeks to more than 9 years), and four trials reported biochemical markers of postoperativebone turnover markers. Table 2 provided outcomes of the 14 including articles.

**Table 1:** Characteristics of the 14 including articles

Study (author/year)	Country	Primary disease	Type of THA	Type of BPs	Control group	Men		Women		Treatment duration		Time of following		Loss to follow-up	
						BPs	control	BPs	control	BPs	control	BPs	control	BPs	control
Tapaninen TS/2010 [18]	Finland	Primary hip osteoarthritis	Uncemented	Alendronate (G_3_)	Calcium	2	5	5	4	0.5 year	0.5 year	5 years	5 years	0	0
Trevisan C/2010 [19]	Italy	NA	Uncemented	Clodronate (G_2_)	No medication	26	27	16	22	1 year	No medication	1 year	1 year	4	8
Arabmotlagh M/2009 [20]	Germany	Degenerative osteoarthritis	Uncemented	Alendronate (G_3_)	No medication	16	9	13	11	5/10 weeks	No medication	6 years	6 years	2	1
Yamasaki S/2007 [21]	Japan	Osteoarthritis secondary to acetabular dysplasia	Uncemented	Risedronate (G_3_)	Placebo	2	2	17	19	0.5 year	0.5 year	0.5 year	0.5 year	3	0
Fokter SK/2006 [22]	Slovenia	Primary or secondary osteoarthritis	Cemented	Etidronate (G_1_)	Placebo	6	3	12	10	1 year	1 year	1 year	1 year	2	1
Arabmotlagh M/2006 [23]	Germany	Degenerative primary osteoarthritis	Uncemented	Alendronate (G_3_)	Placebo	14	12	13	12	0.5 year	0.5 year	52 weeks	52 weeks	0	0
Yamaguchi K/2004 [24]	Japan	Osteoarthritis secondary to hip dysplasia	Uncemented	Etidronate (G_1_)	No medication	5	2	26	22	1 year	No medication	30 months	30 months	2	0
Iwamoto N/2011 [25]	Japan	Osteoarthritis	Uncemented	Alendronate (G_3_)	No medication	4	5	16	17	48 weeks	no medication	48 weeks	48 weeks	2	0
Kinov P/2006 [26]	Bulgaria	Osteoarthritis, osteonecrosis, or hip fracture	Cemented or hybrid	Risedronate (G_3_)	Placebo	4	5	8	7	0.5 year	0.5 year	0.5 year	0.5 year	0	0
Shetty N/2006 [27]	England	Primary or secondary osteoarthritis	Hybrid	Pamidronate (G_2_)	Placebo	12	10	6	9	50 days	50 days	5 years	5 years	1	1
Scott DF/2013 [28]	America	NA	Uncemented	Zoledronate (G_3_)	Placebo + calcium	12	11	15	13	Twice administration	Twice administration	2 years	2 years	0	0
Yukizawa Y/2017 [29]	Japan	Osteoarthritis	Uncemented	Alendronate (G_3_)	No medication	4	7	14	9	≥ 2 years	no medication	≥ 9 years	≥ 9 years	0	0
Muren O/2015 [30]	Sweden	Osteoarthritis	Uncemented	Risedronate (G_3_)	Placebo + calcium	20	18	10	13	0.5 year	0.5 year	4 years	4 years	0	0
Nehme A/2003 [31]	Lebanon	Degenerative hip disease	Cemented	Alendronate (G_3_)	Placebo + calcium	NA	NA	NA	NA	2 years	2 years	2 years	2 years	0	0

*THA* total hip arthroplasty, *BPs* bisphosphonates, *NA* not applicable

### Publication bias

**Fig. 2:**
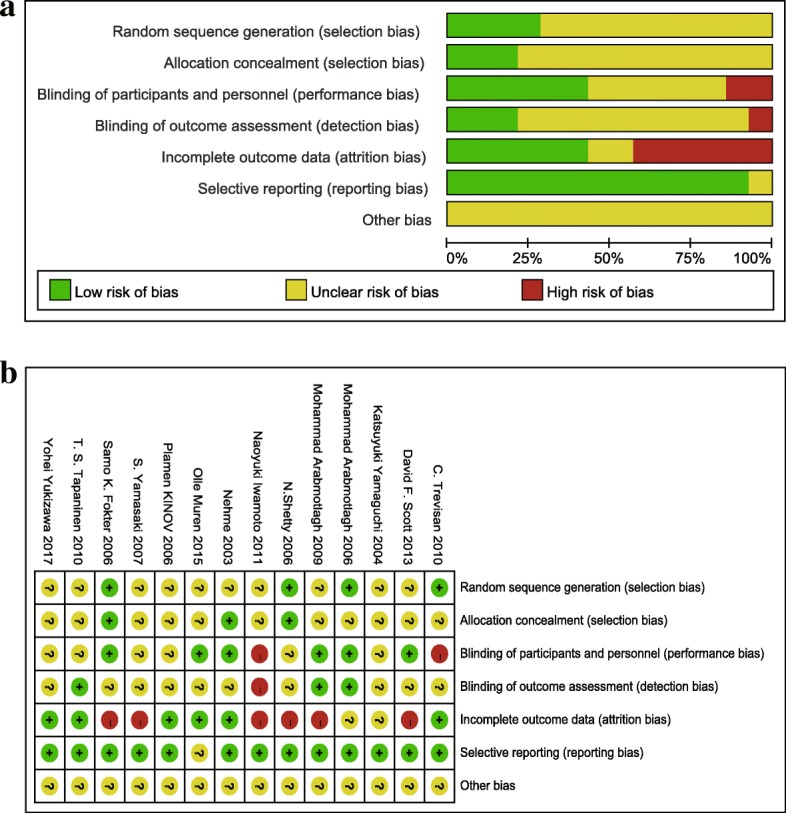
Quality assessment. **a** Risk of bias graph: the author’s judgments about each risk of bias item presented as percentages across all included studies. **b** Risk of bias summary: the author’s judgments about each risk of bias item for all included studies

The quality of included trials was assessed by the Cochrane collaboration’s tool for assessing risk of bias (Fig. 2). All included trials were randomized controlled trials, most of which were low risk of bias and documented randomization, allocation concealment, blinding, and complete outcomes.

**Table 2:** Outcomes of the 14 including articles

Study (author/year)	BMD (mean ± SD) (g/cm^2^)			BAP (mean ± SD) (U/L) (BPs vs. control)	NTX-I (mean ± SD) (nmol/mmol Cr) (BPs vs. control)
	1 year (BPs vs. control)	2–4 years (BPs vs. control)	≥ 5 years (BPs vs. control)		
Tapaninen TS/2010 [18]	− 0.04 ± 0.09 vs. − 0.12 ± 0.10	− 0.05 ± 0.12 vs. − 0.18 ± 0.21	− 0.06 ± 0.12 vs. − 0.16 ± 0.24	NA	NA
Trevisan C/2010 [19]	− 0.04 ± 0.07 vs. − 0.07 ± 0.08	NA	NA	NA	NA
Arabmotlagh M/2009 [20]	− 0.02 ± 0.16 vs. − 0.04 ± 0.09	NA	− 0.02 ± 0.17 vs. − 0.06 ± 0.20	NA	NA
Yamasaki S/2007 [21]	NA	NA	NA	21.5 ± 7.7 vs. 31.2 ± 9.6	39.2 ± 15.9 vs.70.3 ± 27.7
Fokter SK/2006 [22]	− 0.06 ± 0.07 vs. − 0.06 ± 0.23	NA	NA	NA	NA
Arabmotlagh M/2006 [23]	0 ± 0.16 vs. − 0.07 ± 0.22	NA	NA	17.9 ± 6 vs. 27.1 ± 8.9	NA
Yamaguchi K/2004 [24]	− 0.06 ± 0.12 vs. − 0.12 ± 0.14	− 0.09 ± 0.13 vs. − 0.13 ± 0.13	NA	25.2 ± 6.6 vs. 29.6 ± 8.7	52.5 ± 29.2 vs. 71.3 ± 17.8
Iwamoto N/2011 [25]	0 ± 0.12 vs. − 0.08 ± 0.14	NA	NA	NA	NA
Kinov P/2006 [26]	NA	NA	NA	19.93 ± 6.6 vs. 26.9 ± 5.9	NA
Shetty N/2006 [27]	− 0.01 ± 0.07 vs. − 0.02 ± 0.14	− 0.02 ± 0.05 vs. − 0.01 ± 0.16	− 0.02 ± 0.01 vs. − 0.03 ± 0.2	NA	NA
Scott DF/2013 [28]	0.80 ± 10.4 vs. − 6.03 ± 13.2	− 0.16 ± 14.0 vs. − 7.13 ± 12.7	NA	NA	NA
Yukizawa Y/2017 [29]	− 0.04 ± 0.02 vs. − 0.13 ± 0.02	NA	− 0.12 ± 0.03 vs. − 0.21 ± 0.02	NA	NA
Muren O/2015 [30]	NA	− 0.19 ± 0.02 vs. − 0.22 ± 0.03	NA	NA	NA
Nehme A/2003 [31]	− 0.24 ± 0.07 vs. − 0.32 ± 0.07	− 0.09 ± 0.06 vs. − 0.16 ± 0.06	NA	NA	NA

*BPs* bisphosphonates, *BMD* bone mineral density, *BAP* serum bone alkaline phosphates, *NTX-I* urinary N-telopeptide of type I collagen, *NA* not applicable

### Periprosthetic bone mineral density (BMD)

#### BMD at 1 year after THA

**Fig. 3:**
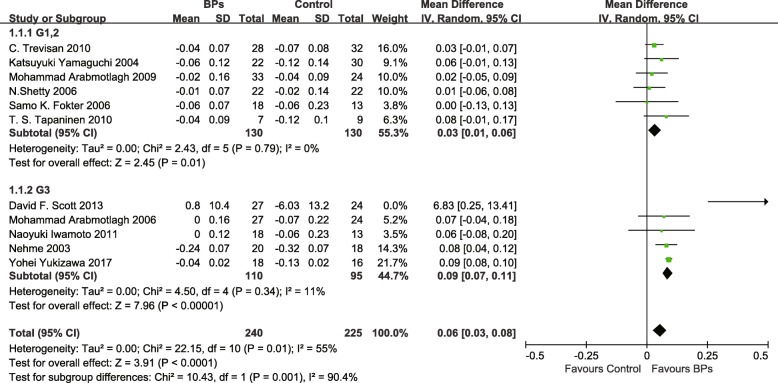
Forest plots showing the effects on BMD at 1 year after THA between BP group and control group

Eleven trials including 465 participants compared BPs with placebo or no medication at 1 year after THA. As showed in Fig. 3, periprosthetic bone resorption in the BP group was significantly less than that in the control group (MD, 0.06 [95% CI, 0.03 to 0.08], *p* < 0.001). Both G 3 BPs and G 1–2 BPs observably inhibit bone resorption, respectively [(MD, 0.03 [95% CI, 0.01 to 0.06], *p* = 0.01); (MD, 0.09 [95% CI, 0.07 to 0.11], *p* < 0.001)]. The difference in BMD between G 3 BP group and the control group was greater than that in between G 1–2 BP group and the control group (*p* = 0.001).

In subgroup analysis, the efficacy of BPs for BMD was significant in the uncemented THA subgroup (MD, 0.05 [95% CI, 0.02 to 0.09], *p* = 0.002), but no significant difference in cemented THA subgroup (MD, 0.06 [95% CI, 0.00 to 0.13], *p* = 0.05). These two subgroup difference was not significant (*p* = 0.76). The duration of BP administration more than 6 months dramatically inhibit bone resorption (MD, 0.07 [95% CI, 0.04 to 0.09], *p* < 0.001). And it seemly obtained more benefit for BMD than the duration less than 6 months, but no difference was showed between the subgroup analysis (*p* = 0.45) (Table 3).

**Table 3:** Subgroup analysis of association between BPs and BMD for each variable

Variable	No. of trials	No. of participants		MD	95% CI	*p* value
		BPs	Control			
1 year after THA						
Type of THA						
Cemented	2	38	31	0.06	0.00–0.13	0.76
Uncemented	9	202	194	0.05	0.02–0.09	
Treatment duration of BPs						
≤ 6 months	5	96	88	0.04	− 0.01–0.10	0.32
> 6 months	6	124	122	0.07	0.04–0.09	
2–4 year after THA						
Type of THA						
Cemented	1	20	18	0.05	0.02–0.09	0.46
Uncemented	4	80	88	0.07	0.03–0.11	
Treatment duration of BPs						
≤ 6 months	4	86	86	0.03	− 0.03–0.09	0.32
> 6 months	2	36	42	0.06	0.03–0.10	
≥ 5 year after THA						
Type of THA						
Cemented	0	0	0	Not estimable	Not estimable	NA
Uncemented	3	54	45	0.09	0.07–0.11	
Treatment duration of BPs						
≤ 6 months	3	54	48	0.03	− 0.03–0.10	0.12
> 6 months	1	18	16	0.09	0.07–0.11	

*THA* total hip arthroplasty, *BPs* bisphosphonates, *MD* mean differences, *CI* confidence interval, *NA* not applicable

#### BMD between 2 to 4 years after THA

**Fig. 4:**
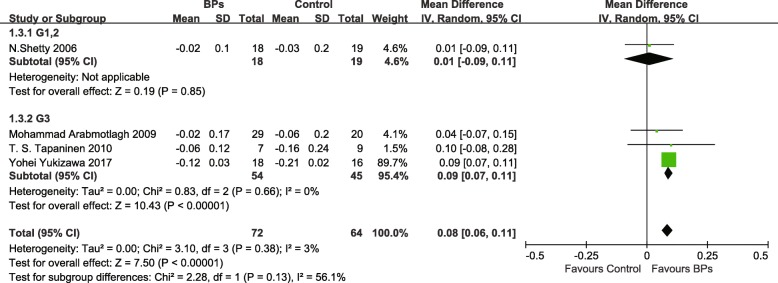
Forest plots showing the effects on BMD between 2 to 4 years after THA between BP group and control group

Six trials including 250 participants compared BPs with placebo or no medication between 2 to 4 years after THA. As showed in Fig. 4, periprosthetic bone resorption in the BP group was significantly less than that in the control group (MD, 0.04 [95% CI, 0.01 to 0.07], *p* = 0.02). G 3 BPs observably inhibit bone resorption (MD, 0.05 [95% CI, 0.01 to 0.10], *p* = 0.03), but not in G 1–2 BP subgroup (MD, 0.01 [95% CI, − 0.04 to 0.06], *p* = 0.69).

In subgroup analysis, the efficacy of BPs for BMD was significant in the cemented THA subgroup (MD, 0.07 [95% CI, 0.03 to 0.11], *p* = 0.0003). But no significant difference was observed comparing uncemented THA group with cemented THA group (*p* = 0.46). The duration of BP administration more than 6 months dramatically inhibit bone resorption (MD, 0.06 [95% CI, 0.03 to 0.10], *p* = 0.0003). However, subgroup difference was not significant on the treatment duration (*p* = 0.32) (Table 3).

#### BMD at more than 5 years after THA

**Fig. 5:**
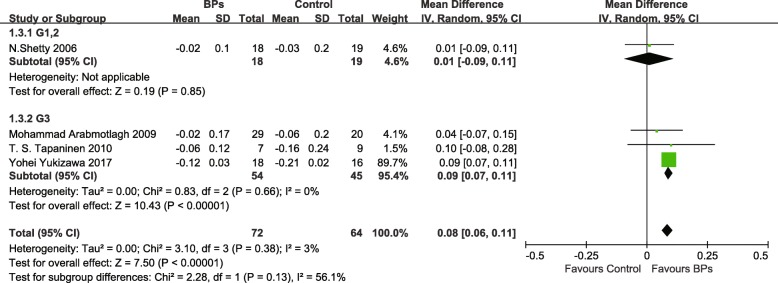
Forest plots showing the effects on BMD more than 5 years after THA between BP group and control group

Four trials including 136 participants compared BPs with placebo or no medication at more than 5 years after THA. As showed in Fig. 5, periprosthetic bone resorption in the BP group was significantly less than that in the control group (MD, 0.08 [95% CI, 0.06 to 0.11], *p* < 0.001). G 3 BPs observably inhibit bone resorption (MD, 0.09 [95% CI, 0.07 to 0.11], *p* < 0.001). No difference was showed in G 1–2 BP subgroup (MD, 0.01 [95% CI, − 0.09 to 0.11], *p* = 0.85).

In subgroup analysis, the duration of BP administration more than 6 months dramatically inhibit bone resorption (MD, 0.09 [95% CI, 0.07 to 0.11], *p* < 0.001). However, subgroup difference was not significant on the treatment duration (*p* = 0.12) (Table 3).

### Serum bone alkaline phosphatase (BAP)

**Fig. 6:**
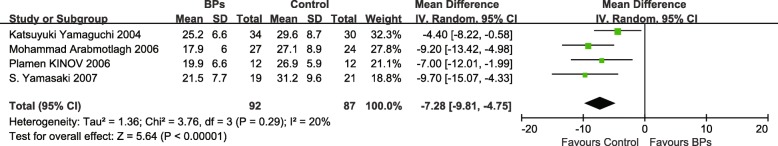
Forest plots showing the effects on BAP between BP group and control group

Four trials including 179 participants compared BPs with placebo or no medication on serum bone alkaline phosphatase. BAP in the control group were significantly higher than that in the BP group (MD, − 7.28 [95% CI, − 9.81 to − 4.75], *p* < 0.001) (Fig. 6). Reviewers did not performed subgroup analyses for BAP as the eligible trials were not enough.

### Urinary N-telopeptide of type I collagen (NTX-I)

**Fig. 7:**
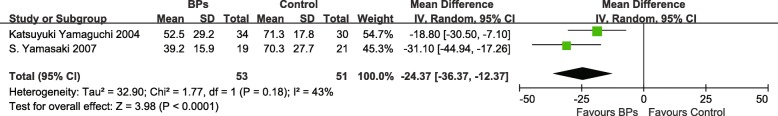
Forest plots showing the effects on NTX-I between BP group and control group

Two trials including 104 participants compared BPs with placebo or no medication on NTX-I. NTX-I in the BP group were significantly lower than that in the control group (MD, − 24.37 [95% CI, − 36.3 to − 12.37], *p* < 0.001) (Fig. 7). Reviewers did not perform subgroup analyses for BAP as the eligible trials were not enough.

## Discussion

This systematic review indicated that BPs could significantly decrease periprosthetic bone resorption at short-, medium-, and long-term observation. The third-generation BPs (G 3 BPs) showed significant efficacy for patients. In addition, this review found that both BAP and NTX-I in the BP group were significantly lower than that in the control group. In subgroup analysis, administration of BPs more than 6 months seemly obtained more benefit for BMD than the duration less than 6 months at long-term observation.

Compared with placebo or no medication, patient in BP group obtained more benefit for BMD especially in the G 3 BP group. Previous studies indicated that aseptic loosening was associated with poor bone quality [32]. The surrounding bone stock provided primary stability of the prosthesis and osseointegration, sealed the bone-implant interfaces, and reduced the implant migration. It was a key factor to avoid aseptic loosening [33]. In the current analysis, BPs could effectively decrease short, medium, and long phase of periprosthetic bone resorption. Besides, low-bone mineral density was a major risk factor for osteoporotic fracture [34]. Meanwhile, the rate of bone loss was an important risk factor for osteoporotic fracture [35]. In our eligible trials, the most of participants were over 50 years old and some of them are postmenopausal women who underwent osteoporosis. Thus, the risk of fracture was high in these participants and it may threaten the longevity of the implant. So BPs may be beneficial for reducing the risk of periprosthetic fracture. In support of us, Alhambra et al. [36] suggested that the use of BPs decreased the fracture risk among THA patients who received BPs as primary prevention (hazard ratio 0.56, 95% CI 0.38 to 0.82) and also among THA patients who had experienced a previous osteoporotic fracture (HR 0.48, 95% CI 0.23 to 0.99).

Base on the present evidence, this study suggested that G 3 BPs were more effective in decreasing periprosthetic bone loss than G 1–2 BPs. Variations in the structure of the side chains determine the strength with which the biphosphonate binds to bone, the distribution through bone, and the amount of time, and it remains in the bone after treatment is discontinued [37]. G 1–2 BPs (etidronate, clodronate, pamidronate, and olpadronate) have simple R2 side chains [38]. Differently, G 3 BPs (alendronate, neridronate, olpadronate, risedronate, ibandronate, and zoledronate) were developed by modifying the R2 side chain to include an amino group and heterocyclic structures, which were found to be up to 1000 times more potent with respect to antiresorptive activity [10]. What is more, G 3 BPs selectively inhibited the cholesterol pathway and subsequently disrupted the osteoclast cytoskeleton with associated osteoclast inactivation [39]. Therefore, G 3 BPs had less effect on osteoblasts and bone formation compared with G 1 BPs [40]. Black and bone also demonstrated the safety of 10 years’ treatment with alendronate for osteoporosis in postmenopausal women [41, 42]. Our result was consistent with it, which also can be applied to inhibit periprosthetic bone resorption.

The significantly lower BAP value in BP group suggested that an influence of BPs may play a role on osteoblast function. Previous studies had found that G 3 BPs had inhibitory effects on terminal differentiation of osteoblasts for bone remodeling, consequently leading to a delay in bone healing [43]. Besides, the unusual mid-shaft long bone fractures were observed in some patients receiving BPs for osteoporosis [44, 45]. Lately, Park et al. demonstrated treatment with BPs more than 5 years was associated with an increased risk of subtrochanteric or femoral shaft fractures [46]. So further investigations were necessary to clarify the duration of BPs or to monitor the bone markers to avoid oversuppression of bone turnover.

With regard to NTX-I, the current analysis suggested that BPs has a strong effect on anti-osteoclast activity. Bone resorption also occurred in the later period, that was focal bone resorption at the prosthesis-bone interface, as a part of the host response to wear debris generated from the prosthesis materials [47, 48]. The wear debris stimulated the release of pro-inflammatory cytokines at the prosthesis-bone interface membrane, the differentiation and activation of osteoclasts, then gave rise to periprosthetic osteolyticlesions [49]. This wear-related osteolysis could also lead to aseptic loosening, which accounting for over 60% of revision surgeries [50]. BPs have been shown promising in reducing osteoclast activity in animal models of particle-induced osteolysis. Shanbhag et al. advocated it for the first time that oral alendronate treatment (5 mg/day for 6 months) could reduce periprosthetic osteolysis in a cementless THA canine model of wear particle-induced osteolysis [51]. Then, Wise et al. further demonstrated that high-dose intravenous zoledronate therapy (10 μg/kg/week) decreased periprosthetic cortical bone porosity and enhanced its mechanical strength in a similar model [52]. In clinical trials, Nishii et al. suggested that alendronate treatment could prevent and restore periprosthetic osteolysis, which was generally thought to require surgical intervention [53].

Bhandari M et al. indicated that BPs presented more efficacies for the cemented group than the uncemented group [16]. However, the report has only included six RCTs of THA and did not conduct any subgroup analysis according to the follow-up time. In the current review, the efficacy of BPs for BMD was significant in the uncemented subgroup at short-term observation, but significant in the cemented subgroup at medium-term observation. Many uncemented implants are larger than cemented implants; thus, stiff stems of uncemented THA may produce more stress shielding and result in greater bone loss at short-term observation [54]. At long-term observation, cemented particles can induce osteoclast differentiation and lead to greater bone resorption compared with uncemented particles [55]. Therefore, the effects of BPs may be magnified by this difference. It may explain BPs worked differently on cemented and uncemented THA. However, only three RCTs were involved in cemented subgroup, which may be difficult to avoid publication bias. To explore the potential efficacies of BPs in different types of THA, more high-quality RCTs were needed.

Administration of BPs more than 6 months seemly obtained more benefit for BMD than the duration less than 6 months. In this subgroup analysis, BMD in more than 6-month group were higher than that in less than 6-month group at all terms of observation. However, the subgroup difference was not significant. These results suggested a significant association of BPs’ long treatment duration with inhibited periprosthetic bone resorption, but the current analysis may lack statistical power to show this association. It was consistent with the previous meta-analysis [17].

Four studies that explored the potential efficacies of BPs have been published [14–17]. However, they had the following limitations: (1) most of them ignored the difference between generation of BPs and did not describe it separately. (2) Target participants in some studies included not only THA but also TKA and hemiarthroplasty. In contrast to previous meta-analyses, this analysis applied more rigorous eligibility criteria and excluded trials involving TKA or hemiarthroplasty to reduce heterogeneity. Furthermore, this analysis not only focused on the efficacies between different generations of BPs, but also discussed effects on treatment duration and types of THA.

Meanwhile, some limitations of this current meta-analysis should be taken into account. First, BMD and biochemical bone turnover outcomes were used to extrapolate the risk of implant revision in this study. However, revision rate in the later follow-up was more objective and ideal. Second, the limited numbers of studies and participants in long-term observation could decrease the strength of our results. Therefore, further RCTs were needed to determine whether a maximum benefit obtainable by BPs, whether benefits increase with increasing duration of administration, whether benefits persist after administration stop, and whether BAP or NTX-I is still suppressed in the later follow-up.

## Conclusion

In conclusion, this study indicated that BPs were beneficial to decreasing periprosthetic bone loss following THA. In short-term observation, G 3 BPs showed greater efficacy for patients.

## Abbreviations

95% CI: 95% confidence interval
BAP: Bone alkaline phosphates
BMD: Bone mineral density
BPs: Bisphosphonates
I^2^: I-square
MD: Mean differences
NTX-I: Urinary N-telopeptide of type I collage
THA: Total hip arthroplasty
